# Intra-operative OCT (iOCT) Super Resolution: a Two-Stage Methodology Leveraging High Quality Pre-operative OCT Scans ★

**DOI:** 10.1007/978-3-031-16525-2_11

**Published:** 2022-09-15

**Authors:** Charalampos Komninos, Theodoros Pissas, Blanca Flores, Edward Bloch, Tom Vercauteren, Sébastien Ourselin, Lyndon Da Cruz, Christos Bergeles

**Affiliations:** 1 School of Biomedical Engineering & Imaging Sciences, King’s College London, SE1 7EU, London, UK; 2 Moorfields Eye Hospital, EC1V 2PD, London, UK; 3 Institute of Ophthalmology, University College London, EC1V 9EL, London, UK

**Keywords:** Image quality, super-resolution, iOCT

## Abstract

Regenerative therapies have recently shown potential in restoring sight lost due to degenerative diseases. Their efficacy requires precise intra-retinal delivery, which can be achieved by robotic systems accompanied by high quality visualization of retinal layers. Intra-operative Optical Coherence Tomography (iOCT) captures cross-sectional retinal images in real-time but with image quality that is inadequate for intra-retinal therapy delivery. This paper proposes a two-stage super-resolution methodology that enhances the image quality of the low resolution (LR) iOCT images leveraging information from pre-operatively acquired high-resolution (HR) OCT (preOCT) images. First, we learn the degradation process from HR to LR domain through CycleGAN and use it to generate pseudo iOCT (LR) images from the HR preOCT ones. Then, we train a Pix2Pix model on the pairs of pseudo iOCT and preOCT to learn the super-resolution mapping. Quantitative analysis using both full-reference and no-reference image quality metrics demonstrates that our approach clearly outperforms the learning-based state-of-the art techniques with statistical significance. Achieving iOCT image quality comparable to pre-OCT quality can help this medical imaging modality be established in vitreoretinal surgery, without requiring expensive hardware-related system updates.

## 1 Introduction

Regenerative therapies (e.g. [20, 6]) have emerged as novel treatment methods for degenerative eye diseases such as Age-Related Macular Degeneration [15], which gradually leads to sight loss. Their success, however, depends on precise delivery to the intra-retinal or sub-retinal space. To this end, alongside novel robotic tools that enable the required implantation precision [5], excellent visualization capabilities are crucial for intra-operative guidance. Intra-operative Optical Coherence Tomography (iOCT) can support such vitreoretinal interventions by providing cross-sectional visualization of the retina and the targeted layers.

In the pre-operative setting, the gold standard for imaging this targeted anatomy, is Optical coherence tomography (OCT), which is a non-invasive imaging modality using infrared light interferometry to visualise retinal layer information. Modern OCT systems use spatiotemporal signal averaging to capture OCT images of excellent quality, enabling clinicians to easily differentiate retinal tissues and layers. However, the long acquisition time during pre-operative OCT scanning makes it unsuitable for the real-time visualization of an intervention. Real-time acquisition is achieved by iOCT albeit at the expense of image quality. More specifically, iOCT images have increased levels of speckle noise [24] and low signal strength [21], which limit their interventional utility. Therefore, we focus on computationally enhancing the quality of iOCT images provided by current commercial clinical systems with the goal of augmenting the capabilities of iOCT technology in the surgical setting without requiring expensive hardware updates.

Image quality enhancement of OCT images has been addressed by various works. Wiener filters [21], segmentation-based [8], registration-based [23] and diffusion-based [2] methods, as well as methods that consider empirical speckle statistics [18], successfully enhanced the OCT quality by reducing speckle noise (denoising) and preserving image structures. However, similar methods can not efficiently be applied on iOCT images and real-time scenarios due to their high computational cost as well as the need of perfect image alignment and prolonged scanning time.

Learning-based techniques using Generative Adversarial Networks (GANs) [9] have been proposed for image quality enhancement or domain translation of natural images [14, 27, 13]. Similar approaches have been adopted for medical imaging modalities such as CT [26], PET [25] and OCT [1, 7, 10]. However, few works have focused on intra-operative OCT image quality enhancement. In [16] iOCT quality was improved using iOCT 3D cubes as the high resolution domain, while in [17] super-resolution achieved through surgical biomicroscopy guidance.

This work concerns self-supervised super-resolution^4^ of iOCT images transferring the quality from high-resolution (HR) pre-operative OCT images to low-resolution (LR) iOCT images. As access to aligned LR-HR pairs is not available, previous approach [17] focused on estimating the HR image of each LR by fusing multiple aligned iOCT video frames and then performing paired super-resolution. Given the fact that their estimated HR images are still of inferior quality compared to preOCT, we propose here a two-stage methodology for the task of unpaired image quality enhancement of iOCT using available preOCT images as HR domain. First, we train a CycleGAN [27] model using iOCT as input and pre-operative, high quality, OCT as the target domain and learn the image degradation process by training the backwards mapping network (HR to LR). Subsequently, the latter is leveraged to generate pseudo iOCT images, which contrary to the starting unpaired dataset, are now aligned with their pre-OCT counterparts. Then, we apply super-resolution with pixel-level supervision through Pix2Pix [13] using the generated pseudo iOCT images. To establish the effectiveness of this approach we provide extensive quantitative analysis showing we outperform existing, state-of-the-art learning based iOCT super-resolution approaches.

## 2 Methods

In this section, we present the data used in our study, the two-stage super-resolution approach and the quantitative metrics used for evaluation.

### 2.1 Datasets

**Fig. 1:**
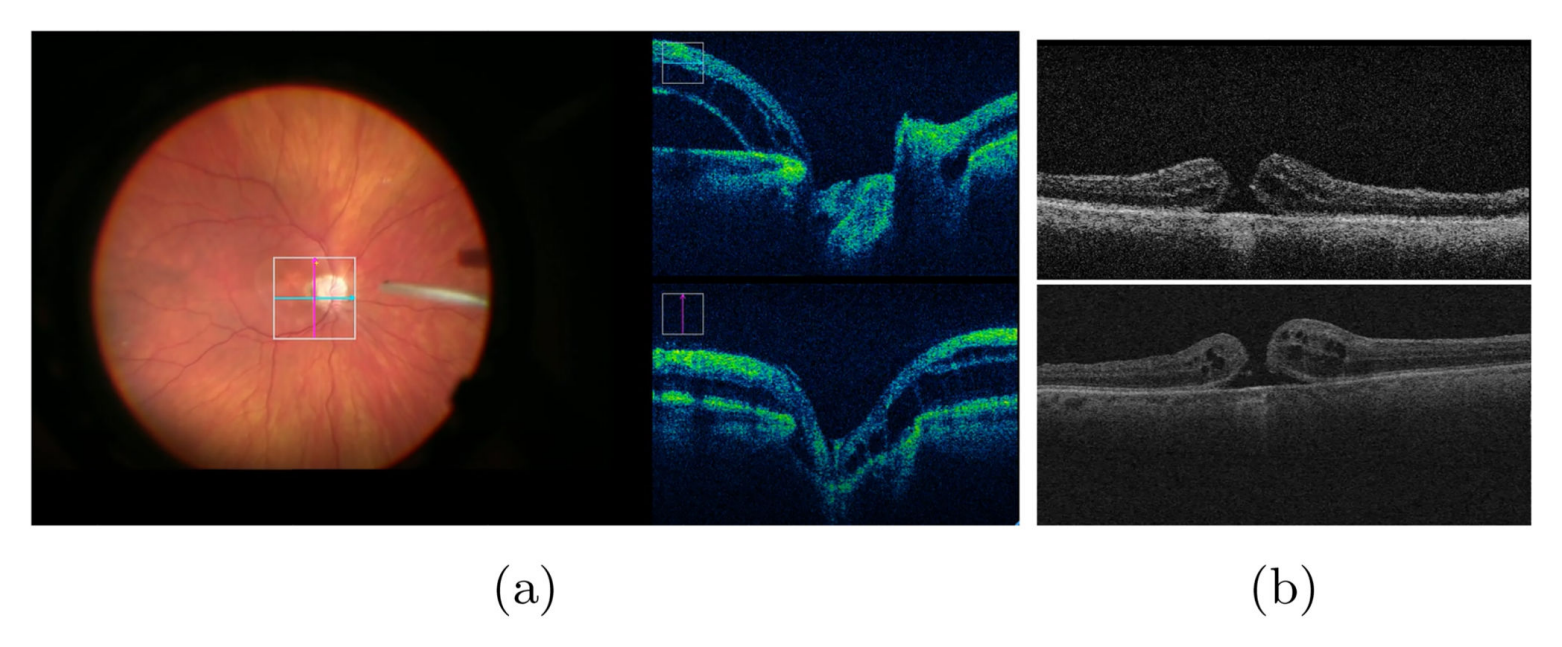
(a): Surgery video frame. Left: Surgical biomicroscope view. Right: iOCT frames. (b): From top to bottom: iOCT and preOCT with macular hole.

The data used in this work are derived from an internal database of intra-operative and pre-operative OCT scans accompanied with vitreoretinal surgery videos acquired at Moorfields Eye Hospital, London, UK (see Fig. 1). The data was acquired in accordance with the Declaration of Helsinki (1983 Revision) and its ethical principles. We use HR pre-operative OCT data (resolution of 512x1024x128 voxels) of 61 subjects which were acquired prior to the surgery using Cirrus 5000 as well as LR intra-operative OCT data (resolution of 440x300 pixels) acquired during the intervention using RESCAN 700 integrated into the Zeiss OPMI LUMERA 700. Pre-operative OCT 2D frames were extracted from the recorded 3D OCT scans.

### 2.2 Two-Stage Super-resolution Approach

**Fig. 2:**
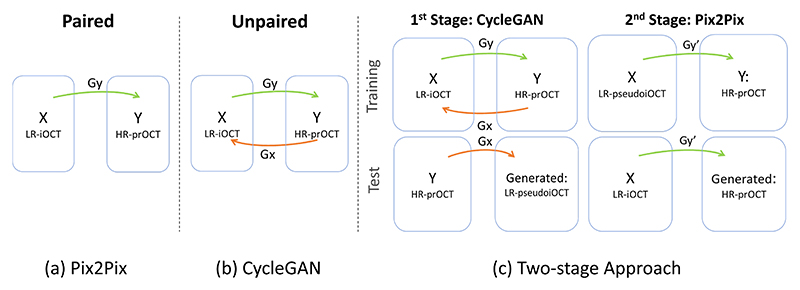
Different approaches for learning the mapping (*G*) between *X* and *Y* domains.

The task addressed in this work is super-resolution (SR) and quality enhancement of iOCT images. Specifically, this task is formulated as domain translation from the iOCT domain to the preOCT domain. In our first attempt, we used CycleGAN’s architecture (Fig. 2.b) as one-stage approach to learn the bidirectional domain translation between HR preOCT and LR iOCT images. However, given that our iOCT and preOCT images are unpaired, and despite the fact that CycleGAN has shown superior performance in unpaired tasks where no pixel-level loss can be employed, as shown in our quantitative analysis it failed to generate consistent results.

We therefore propose a two-stage approach (Fig. 2.c). In the first stage, we use a CycleGAN model to learn the mappings between iOCT and preOCT domains. We leverage the capability of the model to learn with consistency the backwards mapping (from preoCT to iOCT), thus providing a generator that approximates the degradation and domain translation from HR to LR. We then use the trained backwards generator *G*_*x*_ to generate a pseudo (fake) iOCT that is pixel-wise aligned with each real preOCT image. In the second stage, we train a model that learns to map pseudo iOCT images (LR) to the preOCT domain (HR) leveraging pixel-level supervision through the Pix2Pix model. Crucially, as we show in the experimental section, the generator in the second stage sees **only** pseudo iOCT inputs but is able to effectively generalize to real iOCT images.

### 2.3 Implementation Details

The dataset, 7808 pairs of preOCT and pseudo iOCT images, was split into: training set (70%, 43 patients), validation set (15%, 9 patients) and test set (15%, 9 patients). Each patient’s image data were used only in one set. Pseudo iOCT images were generated through the inference of the first stage network achieving similarity of 87.49 Fréchet Inception Distance (see next section) with respect to the real iOCT images.

We based the implementation of the building blocks (Pix2Pix, CycleGAN) of our two-stage approach on the code available online^5^. Both networks use ResNet-based generator [14] of nine residual blocks and are trained on input resolution of 440x300. All models are trained using Adam optimizer with initial learning rate of 10^−4^ and a batch size of 4 for a total of 200 epochs. We used NVIDIA Quadro P6000 GPU with 24 GB memory for our experiments.

### 2.4 Evaluation Metrics

To evaluate the performance of the proposed approach compared to the state-of-the art learning based methods, given that the ground truth HR images do not exist, we use five no-reference Image Quality Assessment (IQA) metrics, i.e. Fréchet Inception Distance (FID) [11], Kernel Inception Distance (KID) [3], perceptual loss function *l*_*feat*_ [16], Global Contrast Factor (GCF) [19] and Fast Noise Estimation (FNE) [12]. FID calculates the distance between distributions of features of two image sets extracted from the ImageNet-pretrained Inception-v3. KID is the squared Maximum Mean Discrepancy between Inception representations extracted from Inception-v3. Perceptual loss *l*_*feat*_ demonstrates how perceptually similar are two image sets by calculating the distance of their representations extracted by Deep Convolutional Network pretrained on Imagenet [22]. GCF calculates the contrast at different resolution levels to calculate the global contrast of the image while FNE measures the noise level of each image of the dataset. We use |*Δ*GCF|, which quantifies the absolute difference of the GCF that SR image yields compared to preOCT, and |*Δ*FNE|, which is the absolute difference of the FNE that SR image yields with respect to preOCT.

Furthermore, we use full-reference metrics following the SR approaches in natural images which apply image degradation techniques to the HR ground truth images to create the LR counterparts. Peak signal-to-noise ratio (PSNR) and Structural Similarity Index (SSIM) are used in our case to evaluate the performance of each model using as input the pseudo iOCT images and comparing its output with real preOCT images.

## 3 Results

In this section, we present the results obtained from the quantitative analysis conducted to evaluate our approach.

### 3.1 Evaluation on real iOCT Images

We compare the improvements in image quality of the iOCT images generated by our model with respect to images from the preOCT domain. A total number of 2352 iOCT frames, extracted from iOCT surgery videos of 9 patients, not present in the train set, was used as test set. As ground truth HR images do not exist, we use five different no-reference IQA metrics, described in 2.4: FID, KID, *l*_*feat*_, |*Δ*GCF| and |*Δ*FNE|.

**Fig. 3:**
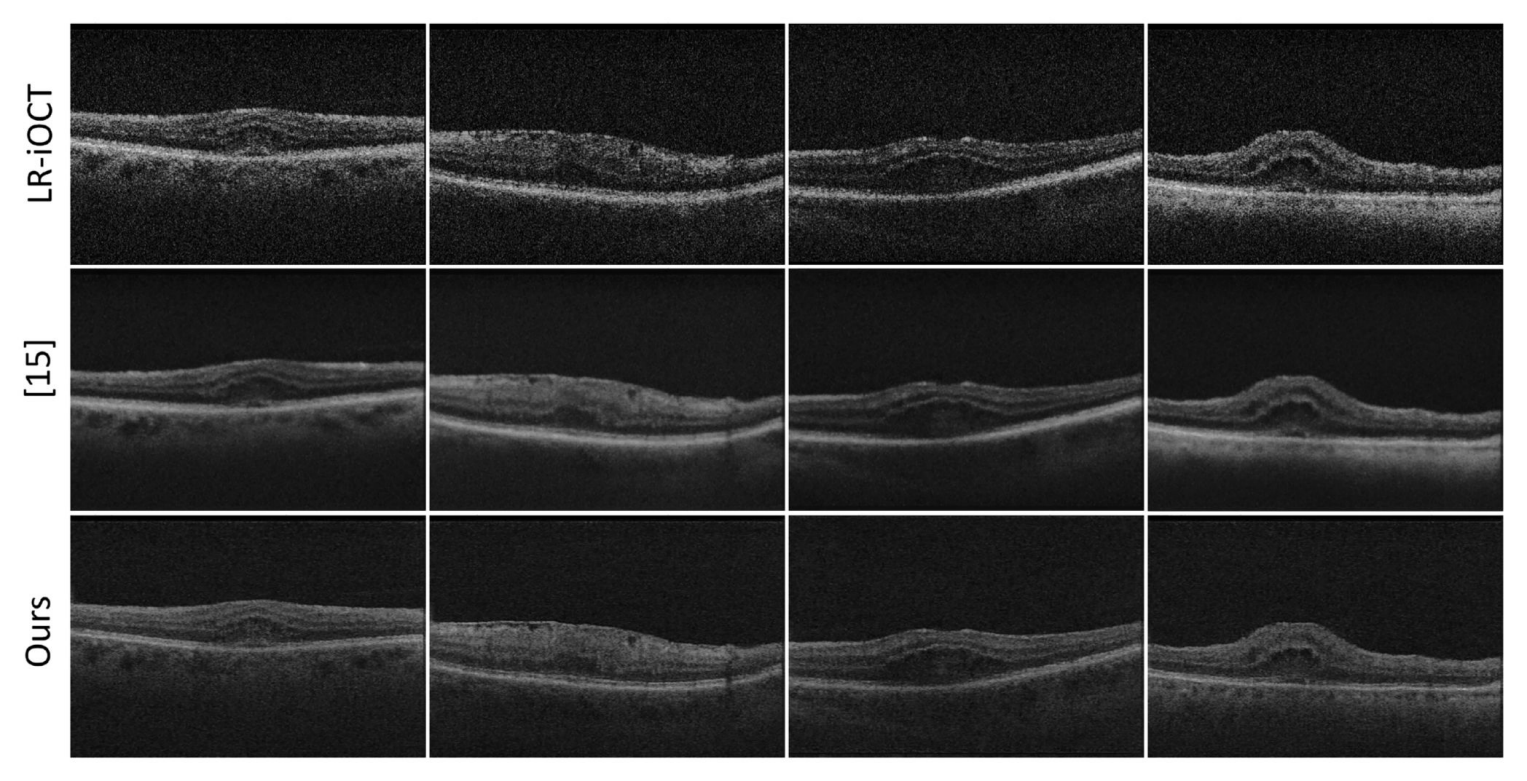
From top to bottom: LR iOCT images, SR using [17], SR using our proposed method.

Table 1 summarises the results of our analysis. Super-resolution using our method ranks first in terms of three out of five no-reference metrics, which demonstrates that the iOCT image quality has been improved (see also Fig. 3) and is closer to preOCT images. Our method is compared to the real iOCT images, the state-of-the-art iOCT SR techniques [16, 17] and the SR using Cycle-GAN with unpaired LR and HR datasets (UnCycGAN). Regarding perceptual metrics, our method exhibit the best FID value and close to the best KID and *l*_*feat*_ values, which demonstrates that our methods can generate SR images that are perceptually more similar to the HR domain. In addition, |*Δ*GCF| and |*Δ*FNE| metrics demonstrate that contrast and noise values of SR images through our method are closer to the values of HR preOCT images, which are the images with the best quality in our dataset. We assessed the statistical significance of the reported values for |*Δ*GCF| and |*Δ*FNE| using a paired t-test and all p-values were p < 0.001. Statistical significance can not be examined for perceptual metrics (FID, KID, *l*_*feat*_) which return one value for all the test set. Furthermore, our approach performs at 18.05 frames per second (FPS) with iOCT images of size 440x300 as input, which is appropriate for the real-time requirement of our application.

### 3.2 Evaluation on pseudo iOCT Images

Prior works [4, 14] used this standard evaluation technique but they applied heuristic degradation processes to generate LR images from their HR counterparts. However, iOCT quality can be affected by speckle noise, low signal strength, different pathologies which are not trivial to simulate. Therefore, we opt for learning the degradation processes. As described in 2.2, during the first training stage we learn the mapping from preOCT to pseudo iOCT and we use it to create pseudo iOCT images that are aligned with our real preOCT for testing, allowing full-reference metrics to be computed.

**Fig. 4:**
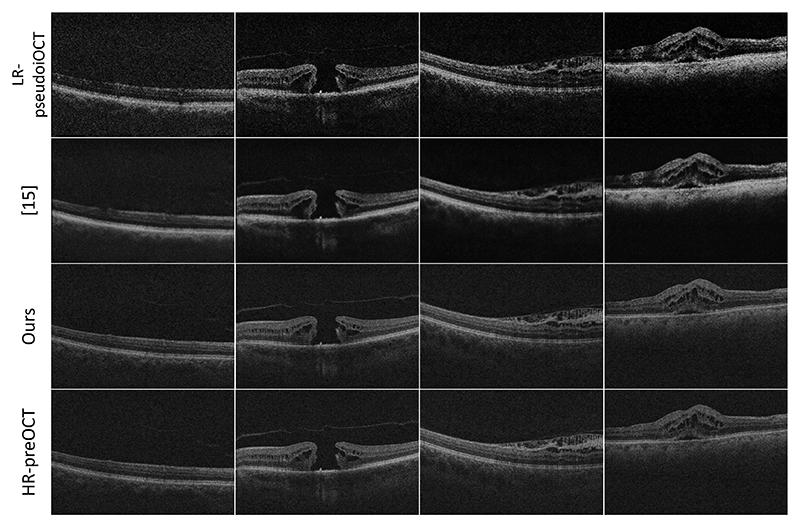
From top to bottom: LR pseudo iOCT images, SR using [17], SR using our proposed method, HR preOCT images.

Thus, quantitative analysis using both full-reference, i.e. PSNR, SSIM, and no-reference metrics was performed on 1152 pairs of pseudo iOCT and preOCT images. The results are reported in Table 2. Our method outperforms all other approaches both numerically and visually as shown in Fig. 4. According to six out of seven metrics, our approach can generate SR images that have high perceptual and structural similarity as well as similar levels of contrast and noise to preOCT images. Paired t-test was used to assess the statistical significance of the pairwise comparisons of the PSNR, SSIM, |*Δ*GCF| and |*Δ*FNE| reported values and all p-values were p < 0.001.

## 4 Discussion and Conclusions

In our study, we propose a Super-resolution pipeline for iOCT images acquired during vitreoretinal surgeries using pre-operatively acquired OCT images as HR domain. Our methodology clearly outperforms both numerically and visually previous proposed image quality enhancement methods.

First, we learn the degradation from preOCT (HR) domain to iOCT (LR) domain through a CycleGAN model trained on unpaired images of the two domains. Then, we apply the learned degradation process to generate pseudo iOCT images from preOCT ones which allows us to create pairs of LR-HR images. Finally, we train a Pix2Pix model on the LR-HR pairs to perform super-resolution.

We quantitatively evaluate our pipeline using as input both iOCT images extracted from real surgery videos and pseudo iOCT images generated through the learned degradation process. The results demonstrate the superior improvement that our method can achieve compared to already proposed techniques.

Future work will include qualitative analysis from expert clinicians and will consider temporal information for the iOCT video super-resolution.

## Supplementary Material

Supplementary material 1

Supplementary material 2

Supplementary material 3

Supplementary material 4

Supplementary material 5

Supplementary material 6

Video_readme

## Figures and Tables

**Table 1:** Quantitative analysis on iOCT images. Arrows show whether higher/lower is better.

	No-Reference				
	FID(↓)	KID(↓)	l*_feat_*(↓)	|*Δ*GCF|(↓)	|*Δ*FNE|(↓)
iOCT	166.06	0.183	527.00	2.33±0.6	7.95±0.6
[16]	171.40	0.191	445.91	2.42±0.5	2.83±0.1
[17]	125.67	**0.115**	435.48	0.85±0.4	3.99±0.1
UnCycGAN	133.33	0.132	**356.48**	0.87±0.3	2.26±0.1
Ours	**123.09**	0.120	379.37	**0.41**±**0.3**	**2.09**±**0.1**

**Table 2:** Quantitative analysis. Arrows show whether higher/lower is better.

	Full-Reference		No-Reference				
	PSNR (*↑*)	SSIM (*↑*)	FID (*↓*)	KID (*↓*)	l*_feat_*(*↓*)	*|*Δ**GCF*|* (*↓*)	*|*Δ**FNE*|* (*↓*)
pseudo iOCT	23.05±2.1	0.65±0.1	121.30	0.114	336.04	1.43±0.5	**2.49**±**0.5**
[16]	16.81±1.8	0.58±0.1	123.18	0.127	362.40	2.48±0.5	3.17±0.1
[17]	24.09±2.2	0.64±0.1	75.43	0.058	277.89	0.41±0.3	4.11±0.1
UnCycGAN	28.93±1.6	**0.82**±**0.0**	58.87	0.041	237.66	0.34±0.2	2.81±0.1
Ours	**31.45**±**0.9**	**0.82**±**0.0**	**16.62**	**0.007**	**76.02**	**0.27**±**0.1**	2.61±0.4

## Footnotes

4 We interchange “super resolution” and “quality enhancement” as usual in the literature.

5
https://github.com/junyanz/pytorch-CycleGAN-and-pix2pix
