# Supplementary material for: Intra-operative OCT (iOCT) Super Resolution: a Two-Stage Methodology Leveraging High Quality Pre-operative OCT Scans
Source: Ophthalmic Med Image Anal (2022). Author manuscript; Available in PMC 2024 Sep 20. (PMC7616592; doi:10.1007/978-3-031-16525-2_11)
Supplement: Video_readme [file EMS197098-supplement-Video_readme.pdf]

This document refers to our submitted work: **"Intra-operative OCT (iOCT) Super Resolution: A Two-Stage methodology leveraging High Quality Pre-operative OCT scans"** and provides details about the videos that we uploaded as supplementary material.

We provide two videos for each of the three subjects (6 in total) that participate in the test set:

1. One of the two videos is named: ***_SR_using_[15]**
   (* indicates the subject number, [15] indicates a different approach that was used for comparison quantitatively and visually in the original paper)
2. The other video is named: ***_SR_using_ours**
   (* indicates the subject number, ours indicates the SR approach that we proposed in the original paper)

Both videos visualize the real iOCT video during vitreoretinal surgeries on the left and the super-resolved video based on different SR approach on the right.
